# Supplementary material for: Gray Matter Abnormalities in Patients with Complex Regional Pain Syndrome: A Systematic Review and Meta-Analysis of Voxel-Based Morphometry Studies
Source: Brain Sci. 2022 Aug 22;12(8):1115. doi: 10.3390/brainsci12081115 (PMC9405829; doi:10.3390/brainsci12081115)
Supplement: Supplementary file 1 [file brainsci-12-01115-s001.zip › brainsci-1719055-supplementary.pdf]

Supplementary Material

Contents

Table S1. PRISMA checklists for systemic review and meta-analysis…………1

Table S2. Systemically search strategy of 3 databases………………………….4

Table S3. Studies quality assessment by 12-point checklist…………………….5

Table S4. Heterogeneity assessment of main results by Q statistics……..…….6

Table S1. PRISMA checklists for systemic review and meta-analysis

Section/topic	Item No	Checklist item	Reported on page No
**Title**			
Title	1	Identify the report as a systematic review, meta-analysis, or both	1
**Abstract**			
Structured summary	2	Provide a structured summary including, as applicable, background, objectives, data sources, study eligibility criteria, participants, interventions, study appraisal and synthesis methods, results, limitations, conclusions and implications of key findings, systematic review registration number	1
**Introduction**			
Rationale	3	Describe the rationale for the review in the context of what is already known	1-2
Objectives	4	Provide an explicit statement of questions being addressed with reference to participants, interventions, comparisons, outcomes, and study design (PICOS)	2
**Methods**			
Protocol and registration	5	Indicate if a review protocol exists, if and where it can be accessed (such as web address), and, if available, provide registration information including registration number	2
Eligibility criteria	6	Specify study characteristics (such as PICOS, length of follow-up) and report characteristics (such as years considered, language, publication status) used as criteria for eligibility, giving rationale	2
Information sources	7	Describe all information sources (such as databases with dates of coverage, contact with study authors to identify additional studies) in the search and date last searched	2
Search	8	Present full electronic search strategy for at least one database, including any limits used, such that it could be repeated	2
Study selection	9	State the process for selecting studies (that is, screening, eligibility, included in systematic review, and, if applicable, included in the meta-analysis)	2
Data collection process	10	Describe method of data extraction from reports (such as piloted forms, independently, in duplicate) and any processes for obtaining and confirming data from investigators	3
Data items	11	List and define all variables for which data were sought (such as PICOS, funding sources) and any assumptions and simplifications made	3

Risk of bias in individual studies	12	Describe methods used for assessing risk of bias of individual studies (including specification of whether this was done at the study or outcome level), and how this information is to be used in any data synthesis	3
Summary measures	13	State the principal summary measures (such as risk ratio, difference in means).	3
Synthesis of results	14	Describe the methods of handling data and combining results of studies, if done, including measures of consistency (such as I2) for each meta-analysis	3
Risk of bias across studies	15	Specify any assessment of risk of bias that may affect the cumulative evidence (such as publication bias, selective reporting within studies)	3
Additional analyses	16	Describe methods of additional analyses (such as sensitivity or subgroup analyses, meta-regression), if done, indicating which were pre-specified	3
**Results**			
Study selection	17	Give numbers of studies screened, assessed for eligibility, and included in the review, with reasons for exclusions at each stage, ideally with a flow diagram	3
Study characteristics	18	For each study, present characteristics for which data were extracted (such as study size, PICOS, follow-up period) and provide the citations	3-4
Risk of bias within studies	19	Present data on risk of bias of each study and, if available, any outcome-level assessment (see item 12).	4
Results of individual studies	20	For all outcomes considered (benefits or harms), present for each study (a) simple summary data for each intervention group and (b) effect estimates and confidence intervals, ideally with a forest plot	5
Synthesis of results	21	Present results of each meta-analysis done, including confidence intervals and measures of consistency	6
Risk of bias across studies	22	Present results of any assessment of risk of bias across studies (see item 15)	7
Additional analysis	23	Give results of additional analyses, if done (such as sensitivity or subgroup analyses, meta-regression [see item 16])	8
Summary of evidence	24	Summarise the main findings including the strength of evidence for each main outcome; consider their relevance to key groups (such as health care providers, users, and policy makers)	9
Limitations	25	Discuss limitations at study and outcome level (such as risk of bias), and at review level (such as incomplete retrieval of identified research, reporting bias)	10-11
Conclusions	26	Provide a general interpretation of the results in the context of other evidence, and implications for future research	11
**Funding**			

Funding	27	Describe sources of funding for the systematic review and other support (such as supply of data) and role of funders for the systematic review	11

Table S2. Systemically search strategy of 3 databases

**Databases**	**Search strategy**
PubMed	((((((((complex regional pain syndromes[MeSH Terms])) OR (Complex Regional Pain Syndrome[Title/Abstract])) OR (CRPS[Title/Abstract])) OR (complex regional pain syndrome, type i[MeSH Terms])) OR (Complex Regional Pain Syndrome, type i[Title/Abstract])) OR (complex regional pain syndrome type ii[MeSH Terms])) OR (complex regional pain syndrome type ii[Title/Abstract])) AND ((((((gray matter[Title/Abstract]) OR (gray matter volume[Title/Abstract])) OR (VBM[Title/Abstract])) OR (voxel-based morphometry[Title/Abstract])) OR (GMV[Title/Abstract])) OR (GM[Title/Abstract]))
Web of Science	(TS=complex regional pain syndromes OR TS=CRPS OR TS=complex regional pain syndrome, type i OR TS=complex regional pain syndrome type ii) AND (TS=gray matter OR TS=gray matter volume OR TS=VBM OR TS=voxel-based morphometry OR TS=GMV OR TS=GM)
MEDLINE	((complex regional pain syndromes or CRPS or complex regional pain syndrome, type i or complex regional pain syndrome type ii).tw.) and ((gray matter or gray matter volume or VBM or voxel-based morphometry or GMV or GM).tw.)

Table S3. Studies quality assessment by 12-point checklist

12-Point Checklists	Barad et al	Domin et al	Pleger et al	Shokouhi et al	Geha et al
**Participants、intervention、comparison**					
★ Patients were evaluated prospectively, specific diagnostic criteria were applied, and demographic data were reported (0 or 1)	1	1	1	1	1
★ Healthy comparison participants were evaluated prospectively; psychiatric and medical illnesses were excluded (0 or 1)	1	1	1	1	1
★Important variables (e.g., age, gender, drug status, and illness duration) were checked either via stratification or statistics (1 or 0.5)	1	1	1	1	1
★All patients were comorbidity free (0 or 1)	1	1	1	1	1
★All patients were medication naive (0 or 1)	0	0	0	0	0
★Sample size per group: ≥ 10; ≥ 20 (1 or 0.5)	0.5	1	1	0.5	1
**Neuroimaging method and analysis**					
★Magnet strength: 3T; 1.5T (1 or 0.5)	1	1	0.5	1	1
★The imaging technique used was clearly described so that it could be reproduced (0.5 or 1)	1	1	1	1	1
★ Whole brain analysis was automated without a previously defined region (0 or 1)	1	1	1	1	1
★Spatial coordinates were reported in a standard space (e.g., Talairach or MNI coordinates) (0.5 or 1)	1	1	1	1	1
**Outcomes and conclusion**					
★ Statistical results were corrected for multiple comparison ; uncorrected (1 or 0.5)	1	1	1	1	1
★ Conclusions were consistent with the results obtained, and the limitations were discussed (0 or 1)	0	1	0	1	0
**Total score**	9.5	11	9.5	10.5	10

Table S4. Heterogeneity assessment of main results by Q statistics

A) Positive peaks

	d	SE	*z*	*P*	CI_{low}	CI_{up}
Barad	1.676	0.432	3.877	0.000105739	0.829	2.523
Pleger	1.370	0.355	3.861	0.000112891	0.675	2.065
Mean	1.493	0.274	5.444	0.000000000	0.956	2.030

Assessment of residual heterogeneity: τ = 0.000, Q = 0.299, df = 1, P = 0.584541321

B) Negative peaks

	d	SE	*z*	*P*	CI_{low}	CI_{up}
Barad	-1.561	0.424	-3.682	0.000231703	-2.392	-0.730
Domin	-1.118	0.289	-3.864	0.000111518	-1.685	-0.551
Shokouhi	-1.359	0.429	-3.170	0.001525699	-2.200	-0.519
Geha	-1.445	0.342	-4.227	0.000023674	-2.115	-0.775
Mean	-1.327	0.178	-7.446	0.000000000	-1.676	-0.977

Assessment of residual heterogeneity: τ = 0.000, Q = 0.951, df = 3, P = 0.813065469
